# The Efficacy and Safety of Multiple Dose Regimens of Kudzu (*Pueraria lobata*) Root Extract on Bone and Cartilage Turnover and Menopausal Symptoms

**DOI:** 10.3389/fphar.2021.760629

**Published:** 2021-10-22

**Authors:** Asger Reinstrup Bihlet, Inger Byrjalsen, Jeppe Ragnar Andersen, Simone Faurholt Simonsen, Kamilla Mundbjerg, Betina Helmer, Bente Juel Riis, Morten Asser Karsdal, Claus Christiansen

**Affiliations:** ^1^ NBCD A/S, Herlev, Denmark; ^2^ Sanos Clinic, Herlev, Denmark; ^3^ Nordic Bioscience A/S, Herlev, Denmark

**Keywords:** pueraria, bone, cartilage, menopausal symptoms, clinical trial

## Abstract

**Background:** Menopause is associated with detrimental changes in turnover of bone and cartilage and a variety of symptoms with negative impact on the quality of life. Naturally occurring isoflavones from *Radix Pueraria lobata*, Kudzu root, may possess chondroprotective and symptom-relieving properties, but efficacy and safety of dosing and dose frequencies required for pharmacological action is unclear.

**Purpose:** This clinical trial evaluates the efficacy on bone and cartilage turnover, menopausal symptoms, and safety of five dose regimens of Kudzu root extract administered either once, twice or three times daily in women with at least mild menopausal symptoms.

**Materials and Methods:** Fifty postmenopausal women were randomized equally into five different dose regimen groups of Kudzu root extract in a four-week, parallel group, open-label, single-center, exploratory study design. Biomarkers CTX-I and CTX-II reflecting bone and cartilage degradation, respectively, were assessed in blood samples and 24-h urine samples. Change from baseline in the Menopause Rating Scale (MRS) and subscales was evaluated. Safety endpoints were frequency of adverse events, changes in hematology and safety chemistry data, vital signs and electrocardiogram.

**Results:** Fifty women (Age 54.2 years, SD: 2.9) were randomized. After 4 weeks of treatment, biomarkers of bone resorption and cartilage degradation were statistically significantly reduced from baseline levels in the group receiving two capsules three times a day, serum/urine CTX-I (−18.4%, 95% CI: −8.1 to −27.5, *p* = 0.001/−34.2%, 95% CI: −21.6 to −44.7, *p* < 0.0001), urine CTX-II (−17.4% 95% CI: −2.5 to −30.0, *p* = 0.02). The observed effects were consistent across study groups but appeared to favour three times daily dosing. Four weeks of treatment led to statistically significant reductions in the MRS Total Score (*p* < 0.0001–0.03) in four out of five treatment groups. Kudzu root extract was well tolerated in all dose regimens, and no serious adverse events were reported.

**Conclusion:** The results indicate that Kudzu extract may possess beneficial effects on bone and cartilage health and may be a promising natural alternative to existing treatments for menopausal symptoms. Kudzu root extract was well tolerated for short-term treatment of mild to severe menopausal symptoms in women in all tested doses and dose frequencies.

**Clinical Trial Registration:** www.ClinicalTrials.gov, identifier NCT04552106

## Introduction

Menopause marks an end of the female fertile period as the result of significantly reduced ovarian production of estrogen and progesterone. As a consequence, women may experience climacteric symptoms with a negative impact on the quality of life (Whiteley et al., 2013) as well as changes in tissue turnover. This directly impactis bone turnover, and may influence joint health e.g. by inducing, or worsening the progression of existing conditions such as osteoarthritis and osteoporosis; both conditions being more common in women after menopause (Bay-Jensen et al., 2012).

Currently the management of post-menopausal osteopenia and joint conditions is limited to the established medical treatment in manifest conditions of osteoporosis and osteoarthritis. Treatment options for climacteric discomfort include local and systemic hormone therapy, which also improves bone health. (Schmidt, 2012; Pinkerton et al., 2017). Systemic hormone therapy is, however, associated with various adverse effects including increased risk of thromboembolic events, breast cancer and a possible association with ovarian cancer, as well as increased risk of endometrial cancer (Pinkerton et al., 2017). This indicates a substantial need for alternatives for the management of menopausal symptoms and associated bone and joint conditions.

*Pueraria montana var. lobata* (Willd.) Maesen & S.M.Almeida ex Sanjappa & Predeep, known as Kudzu, originates from China, the root of which has a history of usage in relation to the treatment of menopausal symptoms as well as conditions affecting menopausal women. The root, due to the content of isoflavones, may possess phytoestrogenic properties (Woo et al., 2003). Kudzu is categorized as a food supplement by the European Commission Health and Food Safety Department (European Commission, 1997). Though Kudzu root has been used for many years in traditional Chinese medicine, evidence regarding dose regimens, their pharmacological profile, effect on bone and cartilage turnover and potential to treat menopausal symptoms of Kudzu extracts is limited (Ulbricht et al., 2015).

Kudzu root is known to contain isoflavones, mainly puerarin and to a lesser extent, daidzein and daidzin. It has been suggested that isoflavones, belonging to the phytoestrogen group of compounds, act similarly to selective estrogen receptor modulators (SERMs) (Treating, 2003) (Lugo et al., 2014). Raw, powdered Kudzu root has been evaluated for effects on menopausal symptoms in an open label trial comparing hormone replacement therapy (HRT) with powdered Kudzu root and no treatment, but with unclear results (Woo et al., 2003). Extraction of root powder may improve the potential for beneficial effects and reduce the amount of powder needed to achieve a physiologically relevant dose. The potential efficacy and safety of Kudzu root extract, its effects on bone and cartilage turnover, dose, and dose frequency necessary to achieve such effects if any, has not previously been adequately researched.

The aim of this study was to evaluate the effects of different doses of Kudzu root extract on bone and cartilage homeostasis, the influence on menopausal symptoms, and safety in women with mild to moderate menopausal symptoms.

## Materials and Methods

The trial was a parallel group, open-label, randomized, single center, exploratory dose-finding study of 4 weeks of treatment in women with at least mild menopausal symptoms (NCT04552106). Study duration was a maximum of 42 days in total with a screening phase of up to 7 days, a treatment phase consisting of 28 days and a follow up phone consultation 7 days after the last day of treatment. Each subject was randomly allocated to one of five treatment groups. The aim was to enroll at least 50 subjects with 10 subjects in each of the following five treatment groups: Two capsules three times daily, three capsules twice daily, two capsules three times daily, two capsules twice daily, or three capsules once daily. The endpoints of the trial were change in the objective biomarkers of bone resorption and cartilage degradation, CTX-I, and CTX-II, respectively, and changes in the Menopausal Rating Scale. The trial endpoints are described in further detail below.

Single daily dosing was administered at a fixed time each chosen by the participant, twice daily dosing administered morning and evening, respectively, and three times daily administration was performed in the morning, afternoon, and before bedtime.

### Kudzu Root Extract

The investigational product was a 0.42 g hard capsule containing 0.28 g freeze-dried ethanol extract from roots of *Pueraria montana var. lobata* (Willd.) Maesen & S.M.Almeida ex Sanjappa & Predeep. The product contained 13.4 g puerarin/100 g and 13.6 g calcium/100 g, corresponding to 37.5 mg of puerarin per capsule. The lowest total daily dose regimen of three capsules was equivalent to 0.84 g of Kudzu extract (corresponding to 113 mg puerarin) and the maximum daily dose of nine capsules corresponded to 2.52 g (corresponding to 338 mg of puerarin).

### Study Population

All participating trial subjects provided written informed consent before any trial procedures were performed. Women in the age between 45 and 60 years considered postmenopausal (defined as at least 12 consecutive months without any menstrual flow) with menopause symptoms, defined as a score of at least 5 out of 44 in the Menopausal Rating Scale (MRS) at screening, were included in the study. Women considered late peri-menopausal (defined as having had a menstrual period more than 3 months ago but less than 12 months ago) were allowed to participate in the study, provided they adhered to use a safe effective method of contraception in the period from consenting until the end of the trial. Due to reported disulfiram-like effects of Kudzu root (Lukas et al., 2013; Penetar et al., 2015), the participants were encouraged to abstain from consuming alcohol during the course of the trial.

Subjects at unacceptable risk of experiencing adverse effects due to past or present significant comorbidities or any clinically significant abnormalities were excluded. Subjects using local or systemic conventional hormone therapy regardless of indication or alternative medication including dietary supplements for the treatment of menopausal symptoms within 28 days prior to randomization were excluded.

### Biomarkers

In order to facilitate comparison of different dose and frequency regimens, 24-h urine samples were collected at home during the entire day prior to each study visit. Assays for type II collagen degradation product uCTX-II, type-I collagen degradation product uCTX-I and sCTX-I were conducted on samples acquired from patients at baseline and each follow-up visit. Serum CTX-I were measured individually by the fully automated Elecsys® electro-chemiluminescent immunoassay analyzers using the S-Crosslaps assay (Roche Diagnostics, GmbH, Mannheim, Germany). Urinary CTX-I and CTX-II was determined using the Urine CrossLaps and Urine Cartilaps ELISAs (IDS Nordic, Herlev, Denmark). Urinary creatinine was measured by a routine chemistry method and used for calculation of creatinine-corrected urinary CTX-I and -II concentrations.

The anticipated pharmacological action of Kudzu is expected to positively affect bone and cartilage metabolism, which allows for an objective exploratory analysis of an efficacious dose using CTX-I and CTX-II as surrogate indicators of pharmacological action. CTX-I and CTX-II were therefore used as indicators of suitable doses of Kudzu extract.

### Menopause Rating Scale

Menopausal symptoms were assessed using the Menopausal Rating Scale (MRS). The MRS is a validated self-administrable standardized questionnaire designed to measure the severity of menopausal symptoms and their impact on the quality of life (QoL). The MRS questionnaire consists of 11 individual questions divided into three subscales; psychological, somatic and urogenital symptoms. Each of the symptoms is graded on a 5-point Likert scale with a minimum score of 0 and a maximum of 4 depending on the severity of symptoms (Heinemann et al., 2004a). The MRS has been shown capable of detecting changes in pre- and post-treatment effects with hormone therapy (Heinemann et al., 2004b) and is considered an appropriate tool to quantify potential effects of Kudzu on menopausal symptoms among the women participating in the study.

The MRS score was obtained at screening to assess the eligibility for study participation, again at baseline and at each clinic visit during the trial period.

### Statistical Analysis

The sample size in this study was not based on power calculations to show statistically significant differences between the treatment arms, but rather to explore trends on safety and efficacy parameters.

Treatment effect on efficacy endpoints was analyzed through mixed-models with repeated measurement (MMRM) on absolute change from baseline or value relative to baseline, including baseline value as covariate, and treatment group, visit, and interaction of visit by treatment group as fixed effects. Least square mean (LSmeans) estimate of change from baseline or value relative to baseline, and pairwise comparisons at week 4 between dose groups were all performed within the context of the MMRM analysis modelling framework. The significance level was set at 5% two-sided for all test. There were no adjustments for multiplicity due to the exploratory nature of the study. Subjects were analyzed according to their randomization group in the following populations; ITT: all randomized subjects, used for subject disposition and demographic and baseline characteristics; modified ITT [mITT]: all ITT patients with baseline and ≥1 post-treatment MRS questionnaire completed, used for efficacy; and safety: subjects were included if they received at least one capsule of Kudzu, used for all safety. All statistical analyses were carried out using SAS version 9.4.

### Safety Endpoints

Adverse event frequency, nature and severity were reported from time of screening throughout the trial period. Clinically significant changes in hematology and safety chemistry data, vital signs and 12-lead electrocardiogram parameters were monitored from screening to the end of treatment.

The study was conducted in compliance with the Helsinki Declaration, and was approved by the National Ethical Committee. The study was funded by Nordic Bioscience.

## Results

### Baseline Characteristics

**FIGURE 1:**
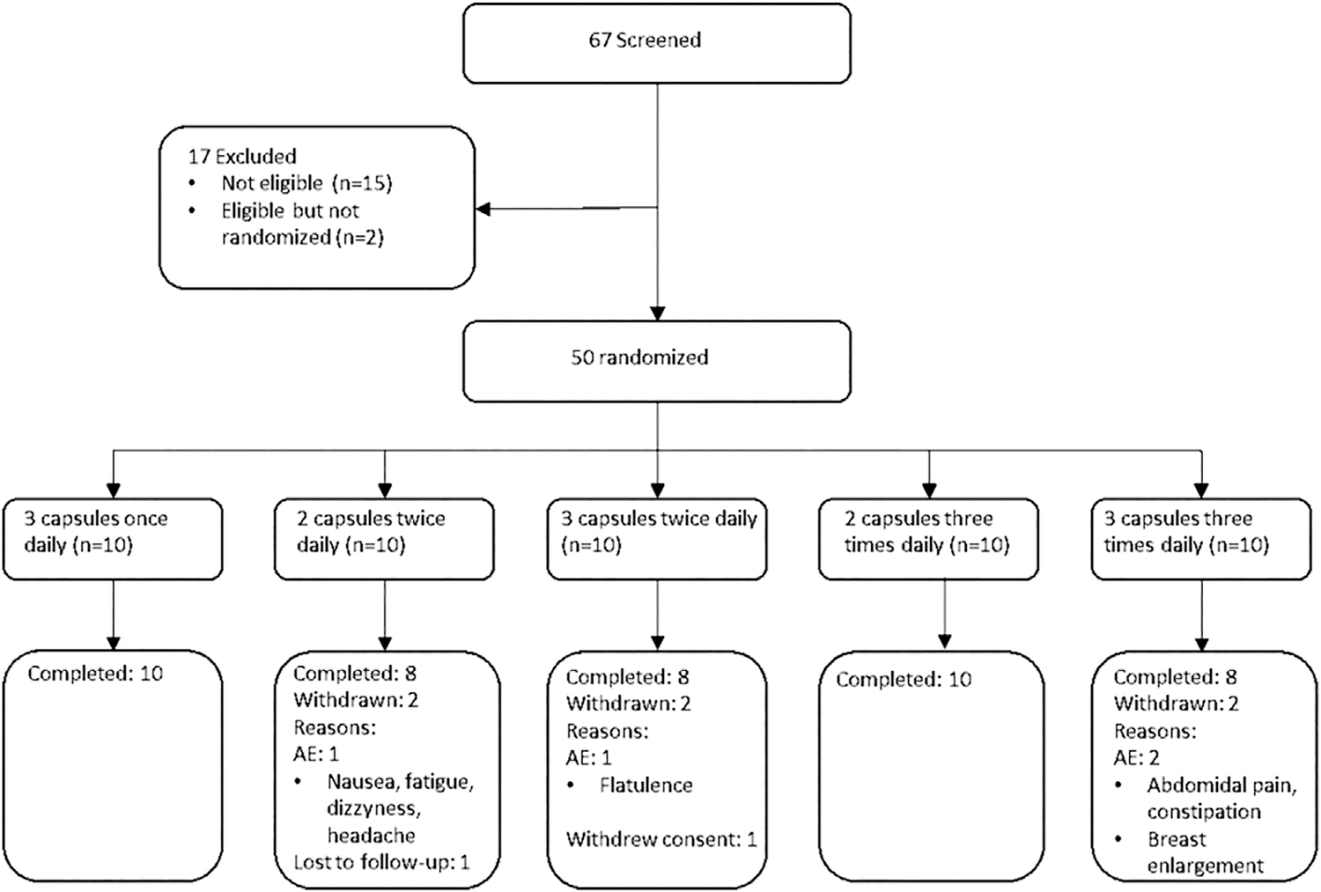
CONSORT flow diagram of participants in the trial.

Sixty-seven female participants were screened in one clinical center, of which 50 women met inclusion criteria and were randomized (Figure 1). The baseline characteristics of each treatment group is shown in Table 1. The treatment groups were comparable with regards to the mean age, BMI, baseline biomarker levels, and baseline MRS score. The mean total MRS score in the pooled study groups was 16.6 (SD = 6.1), out of a maximum score of 44, indicating a mild to moderate symptom severity. The average somatic MRS subscore, evaluating the severity of sweating/flushing, cardiac complaints, sleeping disorders and joint & muscle complaints, was on average slightly higher (mean 7.8 out of 16, SD: 2.5) relative to the subscores of psychological (5.1 out of 16, SD: 3.0) or urogenital symptoms (3.7 out of 12, SD: 2.5).

**TABLE 1:** Baseline demographics, intention-to-treat population.

	Two capsules twice daily (*n* = 10)	Two capsules three times daily (*n* = 10)	Three capsules once daily (*n* = 10)	Three capsules twice daily (*n* = 10)	Three capsules three times daily (*n* = 10)	Total (*n* = 50)
Age, yrs (SD)	55.7 (3.0)	52.8 (3.2)	53.5 (2.0)	54.5 (3.2)	54.4 (2.7)	54.2 (2.9)
BMI, kg/m^2^, (SD)	27.3 (3.4)	28.3 (2.7)	25.9 (2.5)	28.2 (5.3)	24.9 (5.2)	26.9 (4.1)
Total MRS score (0–44), mean (SD)	19.5 (5.7)	14.1 (4.8)	15.5 (6.7)	16.1 (5.9)	17.6 (6.9)	16.6 (6.1)
MRS Psychological subscore (0–16), mean (SD)	6.3 (3.2)	4.4 (2.9)	4.6 (3.0)	5.6 (3.6)	4.7 (2.2)	5.1 (3.0)
MRS Somatic subscore (0–16), mean (SD)	8.4 (2.0)	7.7 (1.8)	7.0 (2.5)	7.9 (2.6)	8.1 (3.5)	7.8 (2.5)
MRS Urogenital subscore (0–12), mean (SD)	4.8 (2.3)	2.0 (1.9)	3.9 (2.5)	3.0 (2.1)	4.8 (2.9)	3.7 (2.5)
sCTX-I, ng/mL (SD)	0.532 (0.183)	0.516 (0.123)	0.553 (0.193)	0.576 (0.191)	0.662 (0.251)	0.568 (0.192)
uCTX-I, µg/mmol (SD)	2.70 (1.60)	2.77 (1.13)	3.33 (1.98)	2.86 (1.23)	3.15 (1.37)	2.97 (1.45)
uCTX-II ng/mmol	255.6 (83.1)	301.6 (204.1)	233.5 (107.2)	303.8 (125.0)	252.5 (95.4)	269.7 (128.7)

### Effects on Bone and Cartilage Turnover

Table 2 displays observed changes in biomarkers of bone resorption and cartilage degradation during the treatment with Kudzu extract.

At Week 4, the urine CTX-I biomarker measured in the 24-h urine samples, was reduced in both groups receiving two capsules three times daily (−34.2%, 95% CI: 44.7 to −21.6, *p* < 0.0001) and three capsules three times daily (−21.4%, 95% CI: 34.4 to −5.7, *p* = 0.01). In the groups receiving two and three capsules of Kudzu twice daily, uCTX-I was reduced at comparable levels (-14.8%, 95% CI: 2.5 to −29.2, *p* = 0.09/(−14.7%, 95% CI: 3.7 to −29.9, *p* = 0.11, respectively). The smallest change in uCTX-I was in the group receiving three capsules once daily (−9.4%, 95% CI: 8.8 to −23.9 *p* = 0.27). The biomarker of cartilage degradation CTX-II measured in the 24-h urine samples, underwent the greatest reduction during the study period in the group receiving two capsules three times daily (−17.4%, 95% CI: −30.0 to −2.5, *p* = 0.02) at week 4. Lesser reductions in uCTX-II were observed in other treatment groups apart from the group receiving three capsules once daily, where no reduction was observed.

The magnitude of decrease in the biomarker values indicate an association between daily treatment frequency, as the groups receiving treatment twice daily appeared to undergo a lower reduction from baseline, as compared to those receiving treatment three times daily, although the differences between treatment groups were not found to be statistically significantly different. The groups receiving once daily treatment with three capsules was shown to reach the lowest reduction in urine CTX-I, and no statistically significant reduction in serum CTX-I and urine CTX-II.

**FIGURE 2:**
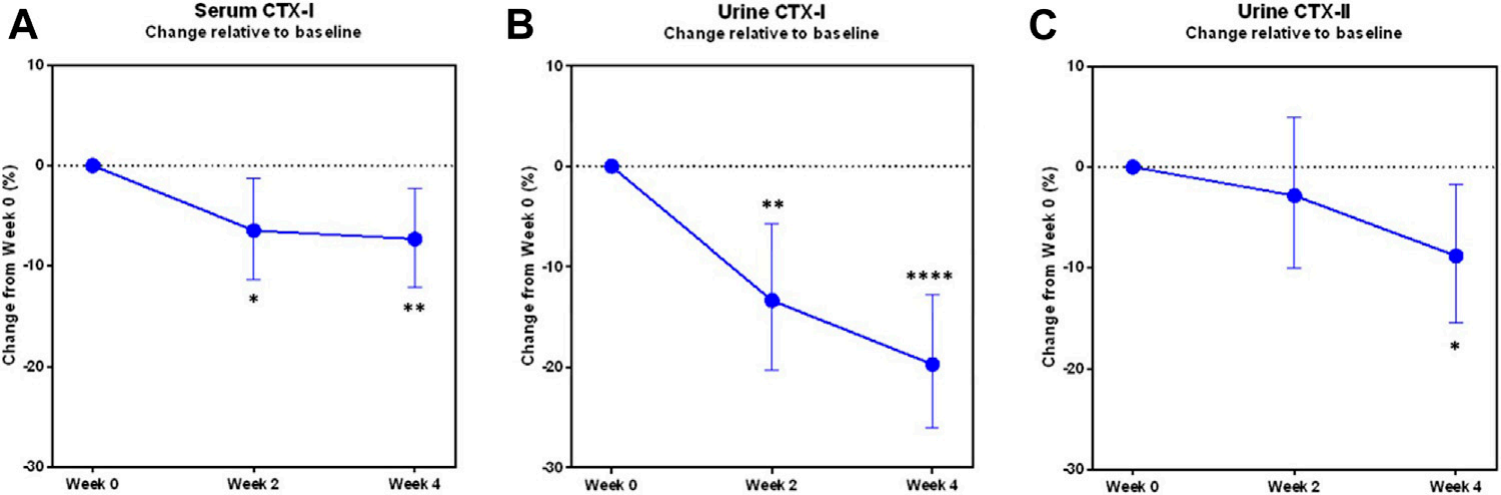
Changes in biomarkers of bone and cartilage turnover during the trial in pooled study groups. The least-square mean proportional changes from baseline in the biomarkers of bone resorption **(A)** sCTX-I, **(B)** uCTX-I, and of cartilage degradation **(C)** uCTX-II, in the modified Intention-to-treat population with pooled study groups (*N* = 49). Error bars indicate the 95% confidence interval. Asterisks indicate statistically significant difference from baseline, *: *p* < 0.05, **: *p* < 0.01, ****: *p* < 0.0001.

Upon pooling of treatment groups, statistically significant changes were seen in all three biomarkers sCTX-I, uCTX-I and uCTX-II after 4 weeks of treatment (Figure 2).

**FIGURE 3:**
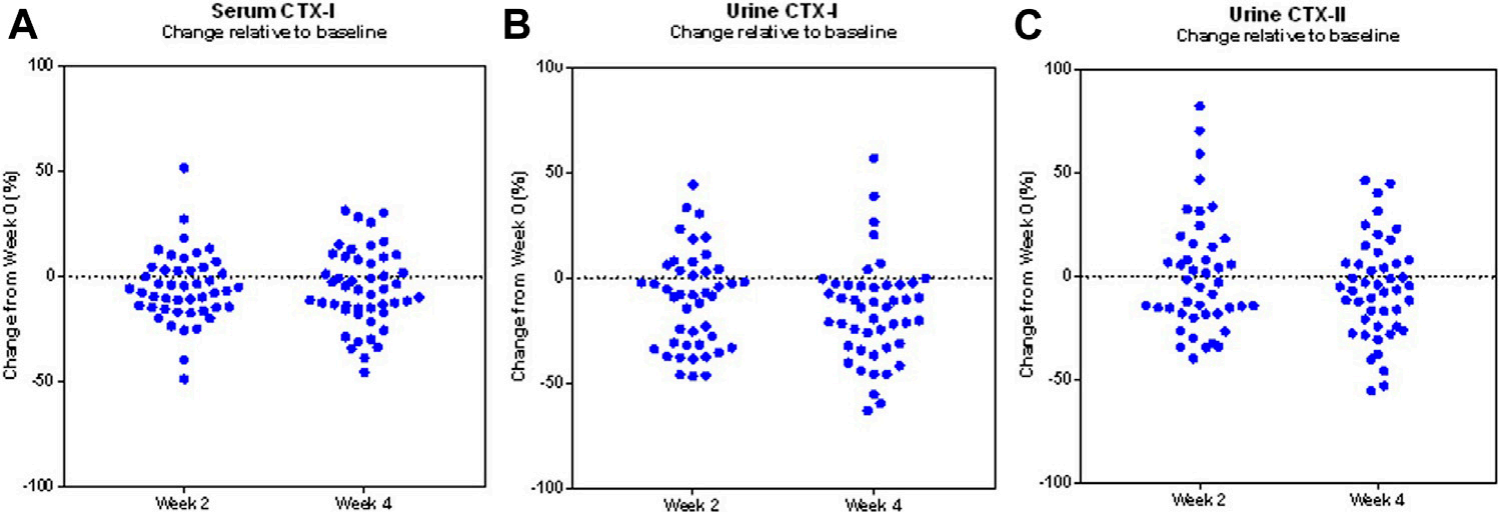
Changes in biomarkers of bone and cartilage turnover in the study population. Scatter plot of the individual proportional changes from baseline in the biomarkers serum CTX-I **(A)**, urine CTX-I **(B)**, and urine CTX-II **(C)**, during the trial in the modified Intention-to-treat population (*N* = 49). Each data point represents the corresponding change from baseline of one study participant.

The changes observed in particularly uCTX-I were consistently favouring a reduction, as shown in Figure 3, as all but six participants experienced a reduction in uCTX-I from baseline during the 4 weeks trial period.

### Effects on Menopausal Symptoms

As shown in Table 2, a statistically significant reduction from baseline in the total MRS score after 4 weeks of treatment was found for all dosing regimens, except for the group receiving two capsules twice daily. The magnitude of change from baseline in the total MRS score ranged from −7.1 out of 44 (95% CI: −4.5 to −9.7, *p* < 0.0001) in the group receiving two capsules three times daily, to −2.4 out of 44 (95% CI: 0.2 to −5.0, *p* = 0.07) in the group receiving two capsules twice daily.

**TABLE 2:** Changes from baseline in biomarkers of bone and cartilage turnover, and Menopausal Rating Scale during the trial.

mITT population	Two caps BID (*n* = 10)				Two caps TID (*n* = 10)				Three caps QD (*n* = 10)				Three caps BID (*n* = 9)				Three caps TID			
	Week 2	p	Week 4	P	Week 2	p	Week 4	p	Week 2	p	Week 4	P	Week 2	p	Week 4	P	Week 2	p	Week 4	p
**sCTX-I, %** (95% CI)	**−6.8** (**−**17.2; 4.9)	0.23	**−9.2** (**−**18.9; 1.7)	0.09	**−9.4** (**−**19.1; 1.5)	0.09	**−18.4** (**−**27.5; **−**8.1)	0.001	**3.3** (**−**7.7; 15.7)	0.56	**1.0** (**−**9.8; 13.1)	0.86	**−5.4** (**−**16.5; 7.2)	0.38	**0.1** (**−**11.2; 12.8)	0.98	**−14.0** (**−**23.6; **−**3.1)	0.01	**−9.0** (**−**18.8; 2.0)	0.10
**uCTX-I, %** (95% CI	**−8.8** (**−**24.8; 10.6)	0.34	**−14.8** (**−**29.2; 2.5)	0.09	**−23.4** (**−**35.7; -8.8)	0.003	**−34.2** (**−**44.7 **−**21.6)	<0.0001	**−8.0** (**−**23.3; 10.3)	0.36	**−9.4** (**−**23.9; 8.0)	0.27	**−5** (**−**22.3; 14.9)	0.57	**−14.7** (**−**29.9; 3.7)	0.11	**-18.0** (**−**31.7; **−**1.7)	0.03	**−21.4** (**−**34.4; **−**5.7)	0.01
**uCTX-II, %** (95% CI)	**0.3** (**−**16.6; 20.5)	0.98	**−10.2** (**−**24.5; 7.0)	0.22	**−6.0** (**−**20.3; 11.0)	0.46	**−17.4** (**−**30.0; -2.5)	0.02	**3.9** (**−**12.8; 23.9)	0.66	**3.5** (**−**12.5; 22.3)	0.69	**−7.6** (**−**23.5; 11.5)	0.40	**−11.4** (**−**26.5; 7.0)	0.20	**−4.6** (**−**19.8; 13.5)	0.59	**−7.9** (**−**22.6; 9.5)	0.34
Total MRS																				
**0–44** (95% CI)	**−2.8** (**−**5.5; **−**0.1)	0.04	**−2.4** (**−**5.0; 0.2)	0.07	**−3.7** (**−**6.3; **−**1.1)	0.005	**−7.1** (-9.7; **−**4.5)	<0.0001	**−2.2** (**−**4.7; 0.4)	0.10	**−6.6** (**−**9.2; **−**4.1)	<0.0001	**−5.1** (**−**7.9 **−**2.3)	0.0005	**−4.5** (**−**7.1; **−**1.8)	0.001	**−3.4** (**−**6.1 **−**0.8)	0.01	**−2.8** (**−**5.3; **−**0.3)	0.03
**MRS Psychological, 0–16** (95% CI)	**−0.9** (**−**2.2; 0.4)	0.19	**−0.2** (**−**1.4; 1.0)	0.71	**-1.5** (**−**2.7; **−**0.2)	0.02	**−2.9** (**−**4.1; **−**1.6)	<0.0001	**−0.5** (**−**1.7; 0.7)	0.44	**−2.0** (**−**3.2; **−**0.8)	0.002	**−1.7** (**−**3.0; **−**0.3)	0.02	**−1.5** (**−**2.8; **−**0.2)	0.02	**−0.9** (**−**2.2; 0.3)	0.15	**−0.8** (**−**2.0; 0.4	0.18
MRS Somatic																				
**0–16** (95% CI)	**−**1.0 (**−**2.3; 0.2)	0.11	**−**1.1 (**−**2.4; 0.1)	0.07	**−**1.3 (**−**2.5;**−**0.1)	0.04	**−**2.7 (**−**3.9; **−**1.5)	<0.0001	**−**0.7 (**−**1.9; 0.6)	0.28	**−**2.9 (**−**4.1; **−**1.6)	<0.0001	**−**2.6 (**−**4.0; **−**1.3)	0.0002	**−**2.4 (**−**3.7; **−**1.1)	0.0004	**−**1.4 (**−**2.7; **−**0.2)	0.03	**−**1.1 (**−**2.3; 0.1)	0.08
**MRS Urogenital**																				
**0–16**	−**1.0** (**−**1.9; **−**0.0)	0.04	−**1.0** (**−**1.9; **−**0.1)	0.03	**−1.1** (**−**1.9; **−**0.2)	0.02	**−1.7** (**−**2.5; **−**0.8)	0.0004	**−0.9** (**−**1.8; **−**0.1)	0.03	**−1.7** (**−**2.6; **−**0.9)	0.0001	**−0.8** (**−**1.8; 0.1)	0.08	**−0.6** (**−**1.5; 0.3)	0.18	**−1.0** (**−**1.9; **−**0.1)	0.03	**−0.9** (**−**1.8; **−**0.0)	0.04

Changes are shown in bold as least-square means. Biomarker data are shown as proportional Least-square means changes from baseline. Menopausal rating scale data are shown as absolute Least-square means changes from baseline BID: Twice daily. TID: Three times daily. *p*-values reflect statistical significance of the change from baseline. mITT: Modified Intention-to-treat. MRS: Menopausal Rating Scale.

In each of the MRS subscales, all treatment regimens except two capsules twice daily and three capsules three times daily led to statistically significant reductions in the respective subscore of the MRS.

### Safety

No adverse changes were observed in safety chemistry, hematology parameters, blood pressure or heart rate during the trial in any treatment group. No serious adverse events were reported during the trial. Adverse events were reported in 90% of the participants, with no apparent dose-relationship. The majority of reported AEs were mild (86%), and no adverse events of severe intensity were reported. The most commonly reported AEs were headache (30%), hot flushes (26%), dry mouth (8%), nausea (10%), and arthralgia (8%), but with no clear dose-relationship (Table 3). Eight individual adverse events in four subjects led to treatment discontinuation (Figure 1).

**TABLE 3:** Adverse events reported. Number and proportion of subjects with adverse events reported with a frequency of more than 5% in the total safety population is shown.

MedDRA system organ class preferred term	2 capsules BID, n (%) (*N* = 10)	2 capsules TID, n (%) (*N* = 10)	3 capsules QD, n (%) (*N* = 10)	3 capsules BID, n (%) (*N* = 10)	3 capsules TID, n (%) (*N* = 10)	Pooled groups, n (%) (*N* = 50)
Constipation	1 (10)		1 (10)		1 (10)	3 (6)
Dry mouth	1 (10)	1 (10)	1 (10)		1 (10)	4 (8)
Flatulence		1 (10)		1 (10)	1 (10)	3 (6)
Nausea	2 (20)	2 (20)			1 (10)	5 (10)
Fatigue	1 (10)		1 (10)	1 (10)		3 (6)
Arthralgia	1 (10)	2 (20)			1 (10)	4 (8)
Headache	4 (40)	2 (20)	4 (40)	4 (40)	1 (10)	15 (30)
Hot flush	2 (20)	5 (50)	2 (20)	3 (30)	1 (10)	13 (26)

## Discussion

The trial constitutes the first clinical trial evaluating the effect on bone and cartilage biomarkers, and safety of different doses and dose frequencies of Kudzu root extract in female subjects with menopausal symptoms.

The current literature on the use of Kudzu root in people with climacteric symptoms lacks important information regarding adequate dosing and dose frequency, and how such regimens affect bone and cartilage. Woo and colleagues performed a community-based randomized trial investigating Kudzu root powder corresponding to a content of 100 mg of isoflavones once daily for 3 months, compared to HRT and no treatment in 136 postmenopausal women (Woo et al., 2003). The study found no evidence of beneficial effects of Kudzu root powder on menopausal symptoms or urine deoxypyridinoline (Dyp) as a measure of bone resorption. These results do not corroborate those of the current trial which demonstrate a consistent reduction in bone resorption as measured by urinary CTX-I in all but six trial participants, reduced cartilage degradation, as well as statistically significant climacteric symptom relief. In comparison, the dose of Kudzu root which was evaluated by Woo et al. may have been too small to reach physiological relevance and did not evaluate other dosing frequencies than once daily dosing in the morning. The present trial evaluated five different dose regimens including three dosing frequencies ranging from 0.84 g of Kudzu root extract to 2.52 g of extract per day, corresponding to 113 and 338 mg of puerarin, respectively per day, compared to 100 mg of isoflavones once daily in the study by Woo and colleagues. For comparison, puerarin is considered to be the major constituent of isoflavones in Kudzu ranging from 19 to 80% of isoflavone content (Wu et al., 2011; Ulbricht et al., 2015). The lack of positive response seen in the Woo study may be explained by the relatively low total daily dose of isoflavones, and the lack of multiple daily dosing as was used in the current trial.

The steady-state elimination half-life of puerarin has been determined to be around 4.5 h (Penetar et al., 2006), and single daily dosing is therefore not expected to provide pharmacological action throughout an entire day. The current results support this, as the results of 24-h urine CTX-I indicate a lesser reduction of CTX-I in the dose group receiving three capsules once daily, as compared to regimens with multiple daily dosing. Additionally, in the group receiving three capsules once daily, serum CTX-I sampling was performed approximately 24 h since the last administration of the investigational product. Considering the known half-life of puerarin, at this point, the bioactive constituents are likely to have been cleared to an extent where suppression of bone resorption is no longer active.

The highest numerical reduction in CTX-I was observed in the group receiving two capsules three times daily. Interestingly, the subject-reported symptomatic relief was highest in the groups receiving three capsules once daily and two capsules three times daily as compared to other regimens, although all regimens except two capsules twice daily led to statistically significant reductions in total MRS score from baseline.

CTX-I (C-Telopeptide of crosslinked Type I Collagen) is the product of the remodeling process of the bones and reflects bone resorption. CTX-I is a well-known biomarker of bone degradation with a high reproducibility and utility in assessing estrogenic properties (Karsdal et al., 2008). As a related process, the degradation of cartilage may be measured by using a similar biomarker. Upon cartilage degradation, extracellular matrix components are being released into circulation and subsequently secreted into urine. The C-Telopeptide of Type II Collagen (CTX-II) is considered as a potential biomarker for cartilage degradation, and urinary CTX-II has been reported to be a useful biomarker of osteoarthritis progression (Kraus et al., 2017; Bihlet et al., 2020). The reduction observed in the bone resorption marker urine CTX-I in the most efficacious dose group; two capsules three times daily, was around 35%, and 20% in serum CTX-I after 4 weeks of treatment. As an objective measure of bone resorption, the observation confirms pharmacological action of the Kudzu extract, however, in the absence of a placebo-group, the current trial did not evaluate the causal pathway associated with this effect. The magnitude of reduction in bone resorption is considered clinically relevant in terms of maintaining bone health, and comparable or higher than that seen with different selective SERMs in women with postmenopausal osteoporosis or osteopenia (Johnell et al., 2002; Silverman et al., 2008; Naylor et al., 2016a), but not reaching the level of that observed using anti-resorptives such as bisphosphonates (Johnell et al., 2002; Naylor et al., 2016b). It should be noted that the changes observed in the current trial were found to be statistically significant from baseline despite the small sample size per treatment group and the relatively short treatment period, which suggests a robust treatment effect on the measured biomarker.

A report investigating puerarin in osteoblast cells found evidence of concurrent activation of osteoprotegerin, the natural decoy receptor of receptor activator of nuclear factor-κB ligand (RANKL), and inhibition of RANKL itself, both contributing to reduced bone resorption. The authors found that these effects were abrogated by an estrogen-receptor antagonist, indicating that the effects are mediated via the estrogen receptor system (Wang et al., 2014). A report by Gray and colleagues found the isoflavone constituents of Kudzu root to be preferentially biased towards the ERβ, but also found differential effects of Kudzu and puerarin alone in assays evaluating sperm motility, indicating involvement of multiple pathways (Gray et al., 2015).

The observation that Kudzu root extract reduced the cartilage degradation marker CTX-II in humans is previously undescribed in the literature. A pre-clinical study of Kudzu extract in ovariectomized rats found similar observations in terms of reductions in CTX-I and CTX-II, indicating reduced bone resorption and cartilage degradation (Luo et al., 2017). This confirmatory finding of the current study has perspectives for the potential of Kudzu root extract to reduce cartilage degradation and thus potentially reduce the risk of development of osteoarthritis or reduce further development of manifest osteoarthritis. The statistically significant reduction in subject-reported MRS subscore of joint and muscular discomfort observed, which was also observed in this trial, supports this hypothesis and warrants further research in an appropriate study population for further investigation.

Investigations of the effects of several phytoestrogens have indicated that high doses may activate the peroxisome proliferator-activated receptor (PPAR) system (Zhi and Lowik, 2005; Dang, 2009; Rietjens et al., 2017)–(Zhi and Lowik, 2005; Dang, 2009; Rietjens et al., 2017), although this observation has not been described with Kudzu root or known Kudzu isoflavones such as puerarin or daidzein alone. Activation of the PPAR system leads to reduced bone formation by biasing bone stromal cells away from osteoblast formation towards increased adipocytogenesis, both of which actions contrast the activation of estrogen receptors. The observation in the current trial that reductions in CTX-I and CTX-II were less prominent in the dose groups receiving higher doses of three capsules either two or three times daily as compared to two capsules three times daily may in part be explained by this phenomenon, as activation of PPAR would counteract the beneficial effects of activated ERs on bone turnover, but the study provides no direct evidence thereof.

### Limitations

The main limitation of the trial is the absence of a placebo-group. The results of subject-reported outcomes including those of menopausal symptoms, are known to be associated with a notable placebo-response. In the absence of a placebo group, the absolute treatment effect on these subjective parameters cannot be determined. The biomarkers as reported in the current report, however, are considered objective and virtually free of placebo-response.

The study did not include a pharmacokinetic measure of circulating isoflavones during the trial, which excludes further analysis of the pharmacokinetic profile of the tested dose regimens. The compliance, as accounted for by manual counting of consumed capsules, was generally very good and was not below 85% for any dose group, which indicates that the assigned dose regimens were consumed as requested. Additionally, the sample size of each treatment group was fairly small, however, the well-established accuracy and reproducibility of the biomarkers used supports the validity of the results in the studied sample size.

As structural changes in bone and cartilage requires a longer observation period to be measurable using imaging techniques, the current study is too short to document any possible changes in bone and cartilage structure.

In conclusion, Kudzu root extract was well tolerated for short-term treatment of mild to severe menopausal symptoms in women in all tested doses and dose frequencies. The results indicate that Kudzu extract may possess beneficial effects on bone and cartilage health, and may be a promising natural alternative to existing treatments for menopausal symptoms.

## Data Availability

The datasets presented in this article are not readily available, however, they may be considered for alternative analysis by the authors upon reasonable request. Requests to access the datasets should be directed to the corresponding author.
